# On detection and assessment of statistical significance of Genomic Islands

**DOI:** 10.1186/1471-2164-9-150

**Published:** 2008-04-01

**Authors:** Raghunath Chatterjee, Keya Chaudhuri, Probal Chaudhuri

**Affiliations:** 1 Molecular & Human Genetics Division, Indian Institute of Chemical Biology, Jadavpur, Kolkata – 700 032, India; 2 Theoretical Statistics and Mathematics Unit, Indian Statistical Institute, 203, B.T. Road, Kolkata – 700 108, India

## Abstract

**Background:**

Many of the available methods for detecting Genomic Islands (GIs) in prokaryotic genomes use markers such as transposons, proximal tRNAs, flanking repeats etc., or they use other supervised techniques requiring training datasets. Most of these methods are primarily based on the biases in GC content or codon and amino acid usage of the islands. However, these methods either do not use any formal statistical test of significance or use statistical tests for which the critical values and the P-values are not adequately justified. We propose a method, which is unsupervised in nature and uses Monte-Carlo statistical tests based on randomly selected segments of a chromosome. Such tests are supported by precise statistical distribution theory, and consequently, the resulting P-values are quite reliable for making the decision.

**Results:**

Our algorithm (named *Design-Island*, an acronym for *Detection of Statistically Significant Genomic Island*) runs in two phases. Some '*putative *GIs' are identified in the *first phase*, and those are refined into smaller segments containing horizontally acquired genes in the *refinement phase*. This method is applied to *Salmonella typhi *CT18 genome leading to the discovery of several new pathogenicity, antibiotic resistance and metabolic islands that were missed by earlier methods. Many of these islands contain mobile genetic elements like phage-mediated genes, transposons, integrase and IS elements confirming their horizontal acquirement.

**Conclusion:**

The proposed method is based on statistical tests supported by precise distribution theory and reliable P-values along with a technique for visualizing statistically significant islands. The performance of our method is better than many other well known methods in terms of their sensitivity and accuracy, and in terms of specificity, it is comparable to other methods.

## Background

Horizontal gene transfer is an important mechanism for the evolution of microbial genomes. In 1990, it was first observed that large blocks of horizontally acquired foreign sequences occur in chromosomes of pathogenic bacteria, and those regions are highly correlated with pathogenicity [1-3]. Some of these possess mobile elements consisting of a gene for specific recombinase and sequences having characteristics of integration sites. Some others, despite their apparently foreign nature, lack insertion sequences, recombinase genes and specific *att *sites, and they may contain only fragments of mobility genes. In the latter case, the mobility sequences were probably lost in course of evolution after their integration into the bacterial genome [1]. The first known foreign DNA blocks that were proved to be associated with virulence genes of pathogenic bacteria were named as *pathogenicity islands *[4]. Later on, genomes of non-pathogenic bacteria have been shown to contain foreign gene blocks, which are not associated with virulence. These gene blocks determine various accessory functions like secondary metabolic activities, antibiotic resistance, symbiosis and other special functions related to the survival in harsh environmental conditions [5]. Subsequently, all foreign gene blocks are collectively named in the literature as genomic islands (GIs) [5,6]. There is an extensive literature on the study of GIs in prokaryotic genomes [7,8]. GIs in prokaryotic genomes often contain horizontally transferred genetic materials as evident from the presence of integrase, transposons, phage mediated genes, etc. in these islands. Consequently, they are critically important in the study of the evolution, the pathogenesis and other special features of prokaryotic genomes.

Several methods have been reported and discussed in the available literature for detecting GIs in prokaryotic genomes [9-13]. Many of these methods use markers such as transposons, proximal tRNAs, flanking repeats etc. to identify GIs [9,11,14]. Mantri and Williams [11] used tRNA and tmRNA as markers. They further searched for the phage integrase and passed through different filtration procedures for the identification of GIs. Ou *et al*. [9] also started with tRNA and tmRNA genes as primary markers, and after passing through different filtration techniques, the GIs were identified. In another previous study, the authors have identified the GIs after performing the cluster analysis of the chromosomal fragments, which are formed by fragmenting the chromosomes based on locations of transposons [14]. Such methods, which are based on standard markers, are particularly useful for detecting GIs acquired by a genome from another compositionally close donor genome or those, which have become compositionally close to the host genome due to the amelioration process. In such cases, the islands may not bear any compositional signature that can be used to distinguish it from rest of the host genome. Consequently, identification of such islands has to rely on possible presence of structural features, like tRNA, direct repeats (DR), integrase gene etc. However, there are limitations of such methods, which are based on standard markers. Firstly, only the GIs, which are associated with standard markers, can be identified by this method. Secondly, there may be intra-chromosomal rearrangements, and islands may no longer be in the proximity of those standard markers after such rearrangements. Consequently, many GIs may not be detected by marker-based methods [7].

In an earlier paper [15], the authors used discriminant analysis, a supervised statistical technique, based on a training data-set that was formed by the authors using the aggregation of known GIs from different organisms. However, unless there are several organisms with some statistical similarities in their genome sequences as well as in their known GIs, such an aggregation to form the training data-set may not be appropriate. Besides, the GIs available and known *a priori *for a single organism may be very few at the beginning of the investigation.

In this paper, we have developed a method that does not use any standard marker when islands are searched in the genome. Islands identified by this method may, however, be confirmed subsequently by supporting factors that include such markers as well as possible presence of known horizontally transferred genes (e.g., phage mediated genes). This will be clear in the section where we discuss the results. Further, the proposed method is unsupervised in nature, and it does not require any training data set for its implementation.

Our method searches for islands in a prokaryotic chromosome using a probing window that slides over the entire chromosome and also varies in its size. For a given size and a given position of that probing window, the segment of the chromosome captured by the window is compared with the rest of the chromosome by means of statistical tests. The outcome of each such test is a statistical P-value that lies between zero and one. A low P-value, which indicates a significant difference between the segments captured by the probing window and the rest of the chromosome, bears evidence for the probing window having a substantial overlap with a GI. All these P-values obtained from statistical tests carried out at different locations and for different sizes of the probing window can be represented by a 3D plot, which enables visualization of locations and sizes of GIs in the chromosome. For the determination of GIs, window based methods have been used in some earlier studies. The GIs of *Pseudomonas putida *KT2440 were determined by analyzing the compositional bias of the mono-, di- and tetra-nucleotide contents in the segment of the genome under the probing window of 4000 bp that slides in steps of 1000 bp [16]. These authors, however, have used windows with fixed lengths, and there is no objective guideline for how to determine that length in practice. Zhang and Zhang [10] used a *windowless *method for displaying the distribution of genomic GC content, and the cumulative GC profile was used by them for the determination of GIs. *Abrupt jump *in cumulative GC profile, which is due to relatively different GC content of an island, enabled them to identify the GI. But this was done in a subjective manner and neither clear quantitative measure nor any formal statistical test for assessing the abrupt change in the cumulative GC profile was proposed by these authors.

Known methods for identifying GIs are primarily based on GC contents of the islands, their oligo-nucleotide usage patterns and the codon usage biases in the genes present in the island [10,12,13,16]. When a fixed segment under the probing window is compared with the whole chromosome, which may contain several GIs (in some cases it might be as large as 20% of the whole chromosome [17]), such a comparison is likely to get influenced by those islands, and this reduces the resolution of the comparison. In order to cope with this problem, we have introduced a *refinement phase *in our algorithm, where the fixed segment under the probing window is compared with randomly selected segments from the chromosome excluding the parts detected as '*putative *GI's' in the *first phase*. This will be discussed in detail in the section on methods.

Various procedures studied in the literature generally lack a formal and rigorous statistical treatment of the problem of comparing a segment of the chromosome with the rest of the chromosome in order to decide whether the segment is the part of a GI or not. Often no formal statistical test is carried out, and the decision to declare a segment as part of an island is done in a subjective way as mentioned earlier. In some other cases, statistical tests have been carried out in a way that is somewhat questionable in the sense that the determination of the critical values and the P-values is not adequately justified due to lack of a rigorous statistical distribution theory of the deviance measures used for such tests. Yoon *et al*. [18] used Mahalanobis distance to evaluate the deviation of the codon usage of a gene from the mean of that in the genome. They assumed normal distribution of codon frequencies without much justification for it, and converted the Mahalanobis distance into a P-value using the χ^2 ^distribution function. They have considered a gene as extraneous in codon usage if its P-value was less than 0.05 [18]. On the other hand, Zhang and Zhang [10] obtained their results based on codon usage and amino acid usage biases using different cut-offs for the P-values. In some earlier studies [19,20], authors used higher order motifs to capture the compositionally deviating regions from the genome. In another study by Vernikos et al. [21], authors used variable order motifs and relative entropy for the detection of compositionally deviating regions. In our method, we have used a Monte-Carlo statistical test, which is partly motivated by the idea of the bootstrap method in statistics [22,23] for comparing the segment under the probing window with randomly selected segments from the rest of the chromosome. Such Monte-Carlo statistical tests based on randomly selected segments of the chromosome can be supported by simple and precise statistical distribution theory.

## Methods

Let us denote a whole chromosomal sequence of an organism by *S*, and *s *will denote a given segment of *S*. In order to assess whether *s *differs significantly from the rest of *S*, we need a measure of distance that can be used for quantitative comparison between the given segment *s *and any other segment *s' *of *S *not having any overlap with *s*. Such a distance measure, which we may denote as *d*(*s, s'*), can be based on GC contents of *s *and *s' *or their oligo-nucleotide distributions. For instance, one may use the absolute distance, the Euclidean distance or Kullback-Leibler divergence computed from oligo nucleotide frequencies. Alternatively, for annotated genomes, one may form the distance measure *d*(*s, s'*) by comparing the gene contents of *s *and *s' *and their codon and amino acid usage biases.

Merkl *et al*. [12] used codon usage analysis of two species assuming the similarity of codon usage in phylogenetically related species. Weinel *et al*. [16] analyzed the di-nucleotide usage and the tetra-nucleotide usage in sliding windows and compared them with the di-nucleotide usage of the whole genome and uniform tetra-nucleotide usage respectively. In the study by Zhang and Zhang [10], putative GIs detected by cumulative GC profile were further analyzed by codon usage and amino acid usage of those regions compared to the whole chromosome *S*. Comparison of the codon usage and oligo-nucleotide usage of the given segment *s *with those for the whole chromosome *S *has some drawbacks because *S *may contain several GIs. In some cases, the total size of the GIs in *S *would be much larger than the length of *s*, and it can be as large as 20% of the size of *S *[17]. This may statistically contaminate values of various parameters related to GC content as well as oligo-nucleotide and codon usage biases when computed for the entire chromosomal sequence *S*. This is likely to reduce the resolution of the comparison. In our algorithm, this issue is carefully addressed by introducing a *refinement phase*, which has been discussed below.

In our method, the comparison between *s *and the rest of *S *is based on *N *randomly selected segments *s*_1,1_, *s*_1,2_, *s*_1,3_, .........*s*_1,*N *_from the chromosome *S*, each of which has the same length as that of *s*, and none of them has any overlap with *s*. We also choose *N *random pairs of segments (*s*_2,1_, *s*_3,1_), (*s*_2,2_, *s*_3,2_), (*s*_2,3_, *s*_3,3_), .........(*s*_2,*N*_, *s*_3,*N*_) from *S*, where for each 1 ≤ *i *≤ *N*, *s*_2,*i *_and *s*_3,*i *_are independently selected, and each of them has the same length as the given segment *s *and no overlap with *s*. Then, we can compute the distances (e.g., distances based on oligo-nucleotide distributions as discussed below) *d*_1,*i *_= *d*(*s*,*s*_1,*i*_) and *d*_2,*i *_= *d*(*s*_2,*i*_, *s*_3,*i*_) for 1 ≤ *i *≤ *N *and form the following two sets of distance values:

*D*_1 _= {*d*_1,*i*_|1 ≤ *i *≤ *N*} and *D*_2 _= {*d*_2,*i*_|1 ≤ *i *≤ *N*}.

If *s *happens to be a part of a GI with characteristics very different from the rest of *S*, the values in *D*_1 _are expected to be larger than those in *D*_2_. Otherwise, the values in the two sets are expected to be of the same order of magnitudes.

### Statistical test for comparing s with the rest of *S*

In view of the way the distance values in *D*_1 _and *D*_2 _have been obtained by random sampling of segments of *S*, the values in each of these two sets can be viewed as independent and identically distributed random variables, and the values in *D*_1 _will be completely independent from the values in *D*_2_. The problem of comparing the values in the two sets *D*_1 _and *D*_2 _can be formulated as a statistical testing problem, where the null hypothesis can be taken as *H*_0_: "the expected value of an element in *D*_1 _is the same as that of an element in *D*_2_," and the alternative hypothesis would be *H*_*A*_: "the expected value of an element in *D*_1 _is larger than that of an element in *D*_2_." We set

$$\begin{array}{cc} m_{1}=N^{-1}\sum_{i=1}^{N}d_{1,i}, & s_{1}^{2}=N^{-1}{\sum_{i=1}^{N}\left(d_{1,i}-m_{1}\right)}^{2}, \end{array}$$

$$\begin{array}{cc} m_{2}=N^{-1}\sum_{i=1}^{N}d_{2,i}, & s_{2}^{2}=N^{-1}{\sum_{i=1}^{N}\left(d_{2,i}-m_{2}\right)}^{2}. \end{array}$$

Then, each of *m*_1 _and *m*_2 _is approximately normally distributed being an average of independent and identically distributed random variables by the well-known central limit theorem in probability theory if *N *is large. Further, *m*_1 _and *m*_2 _are independently distributed, and s_1 _^2^/*N *and s_2 _^2^/*N *will be the standard estimates for their variances respectively. Hence, the statistic

$$Z=\frac{m_{1}-m_{2}}{\sqrt{\left\{\left(s_{1}^{2}/N\right)+\left(s_{2}^{2}/N\right)\right\}}}$$

will be approximately normally distributed for large *N*, and the mean of that normal distribution will be zero if *H*_0 _is true, and it will be positive if *H*_*A *_is true. The variance of that asymptotic normal distribution will be one under both hypotheses. Consequently, *Z *can be used as a test statistic for testing *H*_0 _against *H*_*A *_in a one-sided test. Here, the P-value can be computed using the observed value of *Z *for the given segment *s *under the probing window and the standard normal distribution. This way of assessing the statistical significance of the evidence for *s *being part of a GI in the chromosome *S *using a Monte-Carlo test based on random samples of segments from *S *is partly motivated by the idea of the bootstrap [22,23]. In the present study, we have used *N *= 200. For larger values of *N*, the normal approximation will be more accurate for the distribution of the test statistic, but the corresponding computation time will also increase linearly with *N*, and this might lead to a substantial computational cost when we want to do the analysis for multiple segments with varying sizes located at different positions in the genome. For some smaller chromosomes, we have tried values of *N *up to 500, but the results did not change significantly.

If for some reasons (e.g., computational constraints), one is forced to use smaller values of *N*, the normal approximation for the distribution of *Z *will not be valid. In that case, one may work with a different formulation of the statistical hypotheses as follows. The null hypothesis in that case can be formulated as *H*_0_: "the statistical distribution of an element in *D*_1 _is the same as that of an element in *D*_2_", and the alternative hypothesis can be formulated as *H*_*A*_: "the distribution of an element in *D*_1 _is *stochastically larger *than that of an element in *D*_2_". With these re-formulated hypotheses, one can carry out the test using two-sample Kolmogorov-Smirnov statistic [24] or Wilcoxon-Mann-Whitney statistic [24-26]. These tests, which are computationally more expensive than the test based on normal distribution, have been used by previous authors [14]. However, the power of such non-parametric statistical tests for detecting GIs tends to be less than the preceding test based on normal distribution, which is applicable for relatively larger values of *N*.

### Statistical analysis with segments having variable sizes and locations

In order to identify islands at different locations of the chromosome and to determine the stretches of those islands, it is necessary to carry out our statistical analysis using a probing window that slides across the chromosome and also varies in its size. The statistical test described above can be implemented for any location and size of the segment *s *under that probing window, and the P-value can be computed. It would be useful to plot these P-values so that one can visualize possible locations of the islands in the chromosome as well as their stretches. Such a plot of P-values would also enable us to assess visually the statistical significance of the evidence for or against different segments of the chromosomes to be possible parts of GIs.

For visual presentation of the '*putative *GIs' identified by the analysis described above, a 3D plot for a chromosome can be generated. In this 3D plot, chromosomal locations of the probing window are plotted along the x-axis, corresponding probing window sizes are plotted along the y-axis, and the P-values in gray scale are plotted along the z-axis. Here, the P-value for a specific location and size of the window is plotted using a gray scale that changes gradually from black to white, where black corresponds to the extreme P-value = 0, and white corresponds to the other extreme P-value = 1. The white dots corresponding to higher P-values become almost invisible in the white background while dark dots corresponding to low P-values will be prominently visible marking the '*putative *GIs' in the chromosome.

For a specified value of *P*_0 _(0 <*P*_0 _< 1), one can determine all the segments of a chromosome that are associated with a P-value less than or equal to *P*_0_. This will lead to the identification of some '*putative *GIs' having varying sizes and locations in the chromosome that are identifiable with P-values equal to *P*_0 _or smaller. Ranges of the '*putative *GIs' in terms of their chromosomal locations can be determined using the cut-off value *P*_0 _and considering a specified number of *at least r *overlapping windows of variable sizes having P-values smaller than or equal to *P*_0_.

### Further refinement of the '*putative *GIs' identified by the *first phase *of the algorithm

In the *first phase *of our analysis, the presence of several GIs in the genome may statistically contaminate the randomly sampled segments by affecting their oligo-nucleotide distributions. Besides, '*putative *GIs' obtained using the *first phase *of the algorithm, are always of larger size than what they are supposed to be because of the presence of many 'false positives' (i.e., segments of the genome that are statistically detected as GIs but are not biologically parts of any true island). To reduce the false positives and increase the resolution of our method, a *refinement phase *with a sliding probing window *w *of a fixed size over the regions detected as '*putative *GIs' by the *first phase *of the analysis has been performed. Random samples of genomic segments in the *refinement phase *were chosen from the genome excluding the regions detected as '*putative *GIs' in the *first phase*. This substantially reduces the influence of various possible islands present in *S *on any statistical comparison between *w *and the randomly selected segment, and that in turn increases the resolution of the comparison. The comparison between a probing window *w *and the rest of *S *excluding the regions under'*putative *GIs' is again based on *N *randomly selected segments *w*_1,1_, *w*_1,2_, *w*_1,3_, .........*w*_1,*N *_each of which has the same length as that of *w*. The statistical analysis is very similar to that used in the *first phase*. The P-values are generated using Monte-Carlo tests carried out at variable locations of the probing window with a fixed size.

A smaller probing window is recommended for the *refinement phase *as it will provide a way of precisely detecting the GIs. Gene order conservation is rarely observed in distantly related species and several rearrangements and movement of genes occurs frequently. So, some genes, which are not horizontally acquired from other species, may be present within a '*putative *GI' identified in the *first phase*, and to some extent, this problem is taken care of by the use of a smaller probing window. However, the use of smaller probing window requires randomly sampled segments from non-contaminated stretches of the genome, and those stretches are available after running the *first phase*. Further, the probing window should not be so small that it can be dominated by a single gene, which would increase the effect of codon biases or amino acid biases related to the level of expression or protein function.

Smaller probing windows are not recommended in the *first phase *because it increases the computational cost during the *first phase*. The use of smaller probing windows that slide over the genome lead to a large number of statistical tests, and this may produce many false positive results. Further, there are high chances of substantial overlap of a randomly selected window in the *first phase *with an island in the genome containing horizontally acquired materials.

As in the *first phase *of the analysis, for a specified value of *P*_0 _(0 <*P*_0 _< 1), one can again determine all the segments of a '*putative *GI', which is identified in the *first phase*, that are associated with a P-value less than or equal to *P*_0_.

**Figure 1:**
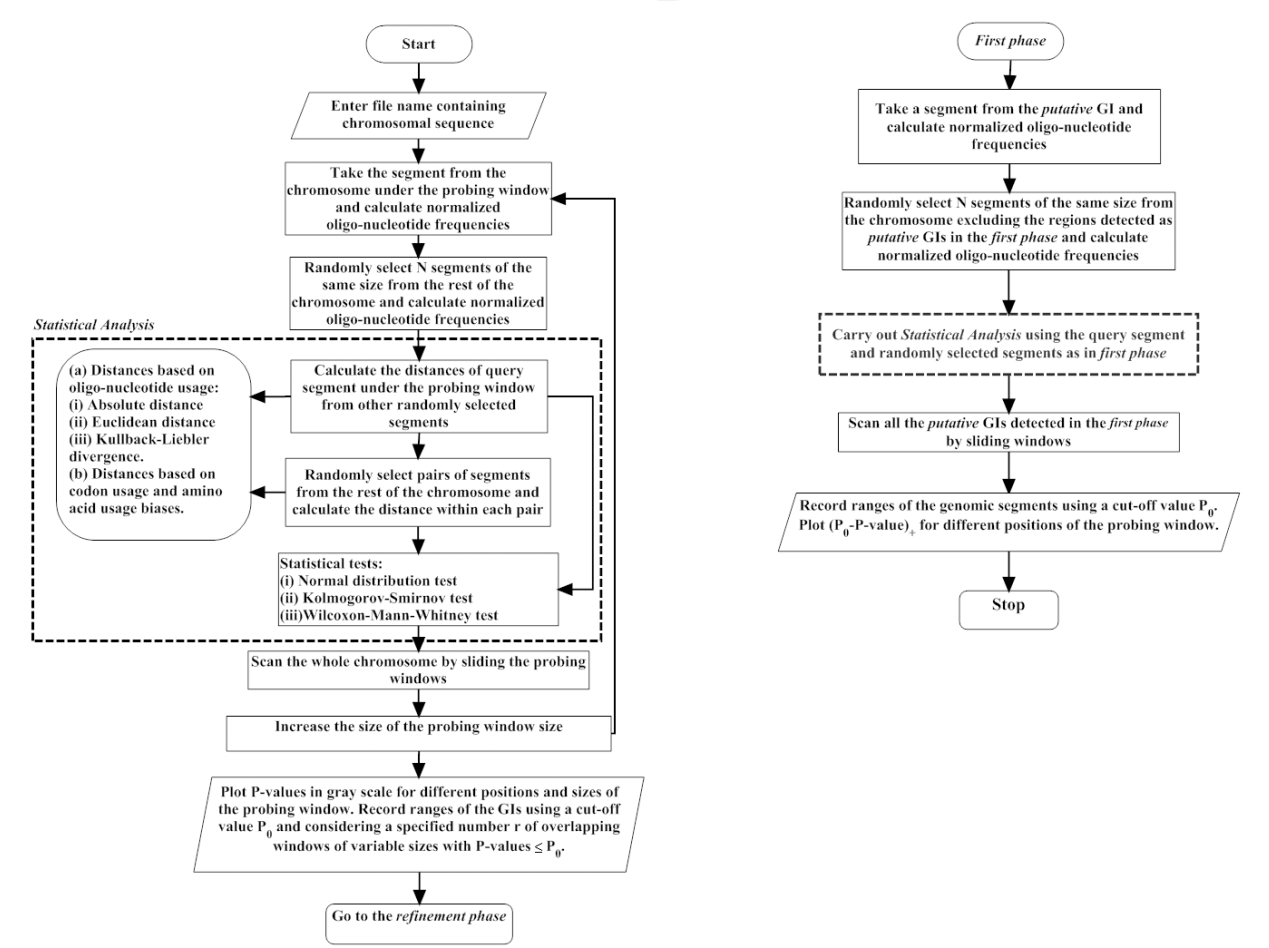
Algorithmic flow-charts of the first phase (Fig. 1A) and the refinement phase (Fig. 1B) of *Design-Island*.

The entire methodology is presented in the form of a flow chart in Fig. 1A, B, and we have named our method as *Design-Island *(an acronym for *Detection of Statistically Significant Genomic Island*).

### Choice of different parameters associated with the algorithm

**Figure 2:**
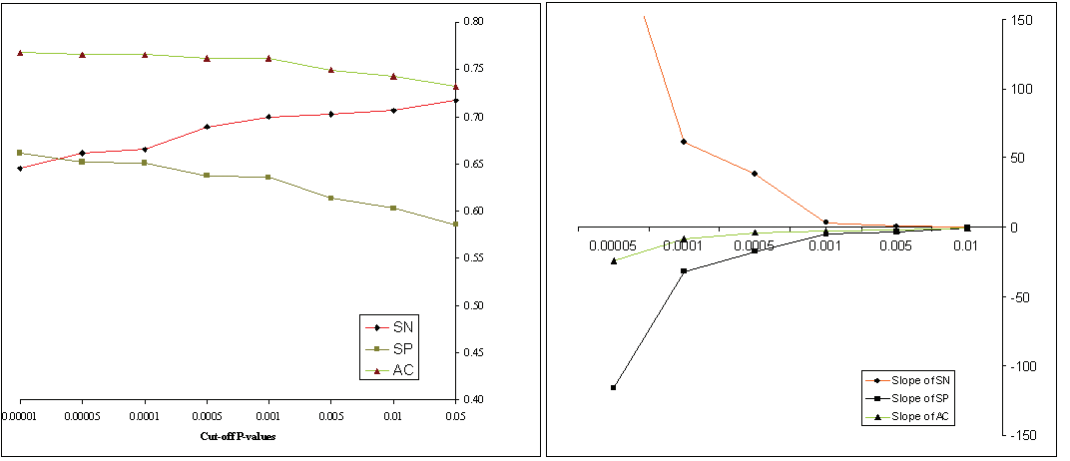
The influence of different choices of cut-off P-values (P_0_) used in the refinement phase on the sensitivity (SN), the specificity (SP) and the accuracy (AC) of Design-Island applied to a manually curated data set of 1560 putative horizontally transferred genes of *Salmonella typhi *CT18 generated by Vernikos et al. [21] is shown in Fig. 2A. Fig. 2B shows corresponding variations of slopes of the curves for SN, SP and AC for different choices of cut-off P-values (P_0_).

In the following section, we have presented results obtained using the absolute distance based on tetra-nucleotide frequencies. Those results are obtained using *P*_0 _= 0.05 and *r *= 5 in *first phase *and *P*_0 _= 0.001 in the *refinement phase*. The value of *P*_0 _in the *first phase *was relaxed to 0.05, and it was chosen in such a way that most of the horizontally acquired stretches of the genome could be captured by the '*putative *GIs' detected in the *first phase*. After we obtain the '*putative *islands', we would be able to generate some statistically non-contaminated stretches of the genome (i.e., genomic regions excluding those putative islands). Those stretches can be used for random sampling of segments in the *refinement phase*. In order to determine the value of *P*_0_ in the *refinement phase*, we have carried out a performance assessment of our method for different values of *P*_0 _based on a dataset related to *Salmonella typhi *CT18 generated by Vernikos et al. [21]. Their method of constructing the dataset of putative horizontally transferred genes is discussed briefly in the section on results and discussion. We have calculated the sensitivity (SN), the specificity (SP) and the accuracy (AC) of our method for different values of *P*_0 _ranging from *P*_0 _= 0.05 to *P*_0 _= 0.00001 (Fig. 2A). The slopes of the curve for SN, SP and AC were also plotted for different values of *P*_0 _(Fig. 2B). As this cut-off P-value increases, the specificity and the accuracy increase, but the sensitivity decreases. We have observed that the specificity and the accuracy increase steadily up to *P*_0 _= 0.001 (Fig. 2A), and then the slope of each of the two curves decreases (Fig. 2B). The sensitivity was observed to decreases with the increase in the cut-off P-value, but in the region from *P*_0 _= 0.05 to *P*_0 _= 0.001, the sensitivity decreases slowly, and then it decreases much more sharply. Alternatively, one can determine the value of *P*_0 _using the ROC curve approach. When we used that technique with a range of *P*_0 _values from 0.05 to 0.00001, it again led to the same value of *P*_0 _as the optimal, and we have chosen the cut-off P-value as *P*_0 _= 0.001 for the *refinement phase*. It is possible that for some other bacterial genomes, a different choice of *P*_0 _would be optimal depending on the nucleotide compositions of those genomes. However, some empirical studies using this choice of *P*_0 _for some bacterial genomes other than *S. typhi *CT18 demonstrated reasonable performance of our algorithm.

We have carried out our analysis with distance measures based on oligo-nucleotides of different orders (i.e., sizes). The islands detected by methods based on different orders of oligo-nucleotides did not differ considerably. Only in some cases either the boundaries of the segments of the '*putative *GIs' slightly differed or a single '*putative *GI' broke into two or more segments. In most of the organisms, the '*putative *GIs' detected using tetra-nucleotide analysis include those detected by other analysis based on other oligo-nucleotides, and the later analysis sometimes missed some of the important segments of the genomes containing known horizontally acquired materials. As we will see in the section containing a comparative study of different methods, our method outperformed the method W8 [20], which is a method based on octa-nucleotides, in many cases.

We have considered three types of distance measure computed using oligo-nucleotide frequencies. These are the absolute distance, the Euclidean distance and the Kullback-Leibler divergence. But all these distances lead to almost the same result. The '*putative *GIs' detected by methods based on different distances tend to differ in their boundaries to a small extent. We have finally decided to use the absolute distance, which is computationally the simplest among all the distances considered. Before computing the distances, as some authors suggested [27-32], one may normalize higher order oligo-nucleotide frequencies by lower order oligo-nucleotide frequencies based on Markov type models.

One may, in principle, use distances computed using codon usage or amino acid usage biases instead of oligo-nucleotide distributions. However, that will require the use of complete annotation of the entire chromosome and the gene content of each and every randomly selected segment for our Monte-Carlo test. This makes the implementation of the method computationally challenging, and we have not pursued that here.

## Results and Discussion

We have implemented *Design-Island *on the chromosome of *Salmonella typhi *CT18 obtained from NCBI database [33]. The co-ordinates of statistically significant genomic segments detected by *Design-Island *and their gene contents in the chromosomes of *S. typhi *CT18 are presented in Additional file 1 and detected segments of *Salmonella typhi *CT18 are discussed below.

### *Salmonella typhi *CT18

*Salmonella enterica serovar Typhi *(*S. typhi*), an aetiological agent of typhoid fever, is a serious invasive bacterial disease of human. Many *S. enterica serovars *actively invade the mucosal surface of the intestine but are normally contained in healthy individuals by the local immune defence mechanism. However, *S. typhi *has evolved the ability to spread to the deeper tissues of human including liver, spleen and bone marrow [34]. In *S. typhi*, thirteen pathogenicity islands (popularly known as SPIs – *Salmonella *Pathogenicity Islands) and five islands containing bacteriophages related genes have been reported [21,34].

In *S. typhi *CT18, *Design-Island *detected ninety seven '*putative *GIs' in the *first phase*, and after refinement, these islands are broken into two hundreds and twenty-one statistically significant genomic segments that include all of the GIs detected in the previous studies. Major genes contained in these segments code for phage proteins, putative pathogenicity island proteins, virulence associated secretory protein, Vi polysaccharide proteins, integrase, phage integrase, putative bacteriophage proteins, IS element transposases, flagellar proteins, UV protection protein, type III secretion system, type III restriction-modification system, killing factor KicA and B, different chains of NADH dehydrogenase and heat shock proteins. Among the newly detected genomic segments, the major genes present are those, which code for putative toxin like proteins, putative virulence proteins, putative phage proteins, integrase, type III restriction modification system, some pseudo genes, some transporters, flagellar biosynthetic proteins and several accessory proteins, different chains of NADH dehydrogenase and ATP synthase, penicillin binding protein, fimbrial subunits, lipopolysaccharide core biosynthesis protein, heat shock and cold shock proteins.

**Figure 3:**
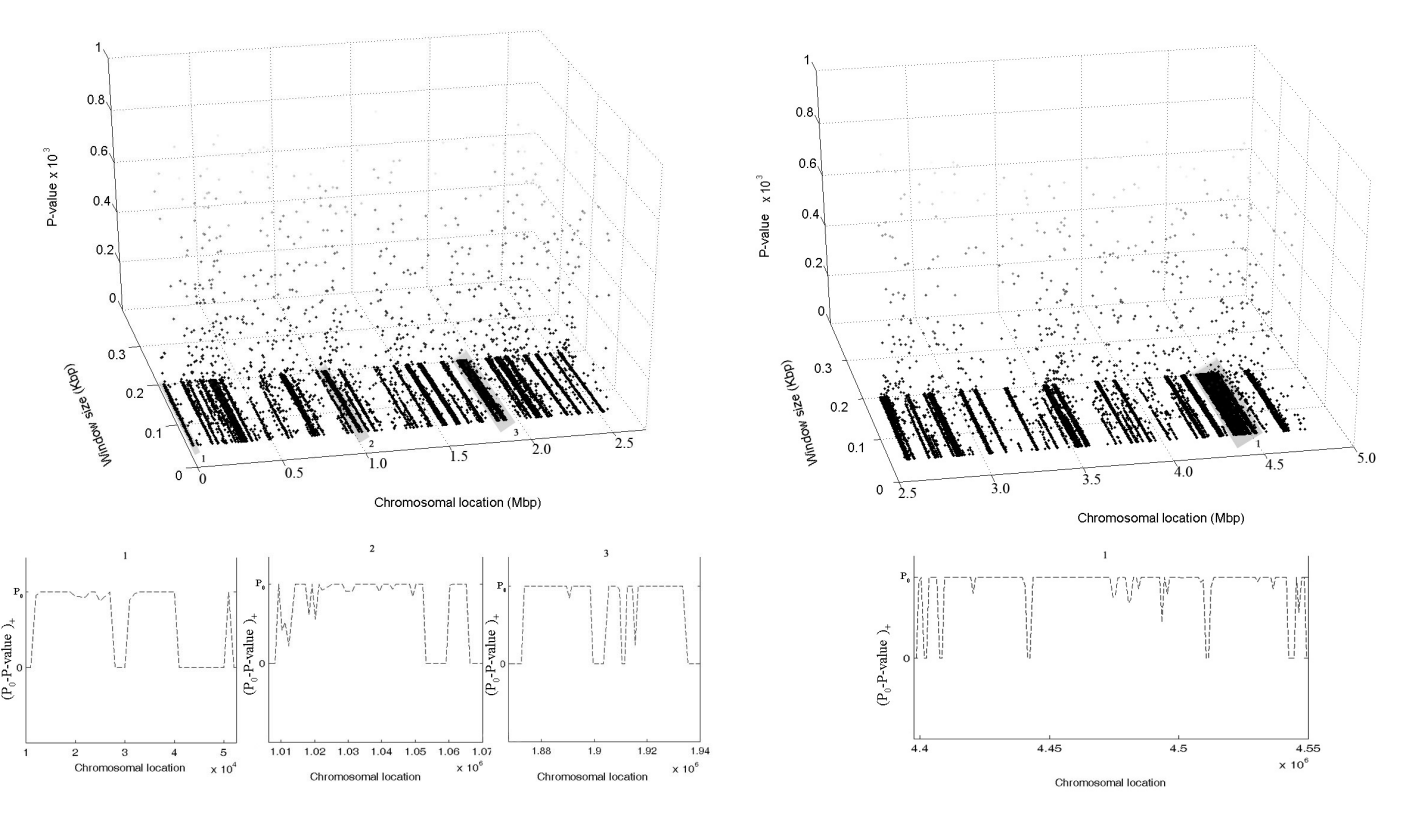
In the upper panel, 3D plots of the P-values for a window with variable size that slides across (i) the chromosome of *Salmonella typhi *CT18 from 1 bp, i.e., the start to 2.5 Mbp (Fig. 3A), (ii) the chromosome of *Salmonella typhi *CT18 from 2.5 Mbp to 4.8 Mbp, i.e., end (Fig. 3B). The P-value at a specific location and for a specific size of the window is plotted using a gray scale that changes gradually from black to white with black corresponding to the extreme P-value = 0 and white corresponding to the other extreme P-value = 1. The white dots corresponding to higher P-values are almost invisible in the white background while dark dots corresponding to low P-values are prominently visible marking the GIs in the chromosome. Lower panel in each figure gives some representative 1D plots generated from the refinement phase for some of the 'putative GIs' (enclosed in gray blocks and labeled as 1,2,... in the 3D plots) detected in the first phase of Design-Island. The quantity (P_0_-P-value)+ for the region of a GI detected in the first phase is plotted. Here, for P-value > P_0_, (P_0_-P-value)+ = 0, and for P-value < P_0_, (P_0_-P-value)+ = (P_0_-P-value).

Two 3D plots generated from the *first phase *of our algorithm and some representative 1D plots generated from the *refinement phase *of the algorithm applied to the chromosome of *S. typhi *CT18 are shown here in Fig. 3A, B. The first plot corresponds to the stretch of the chromosome from the start of the chromosome up to 2.5 Mbp position (Fig. 3A), and the other plot corresponds to the stretch of the chromosome from 2.5 Mbp position up to the end (Fig. 3B). Representative 1D plots for four of the '*putative *GIs' detected in the *first phase *and enclosed in gray blocks are shown in the lower panel of the figures. The '*putative *GI' that stretches from 10000 to 52500 is fragmented into three segments, namely 11000–28000 bp, 30000–41000 bp and 50000–52000 bp. The '*putative *GI' that stretches from 1006250 to 1070000 bp is fragmented into two segments, namely 1008250–1053250 bp and 1059250–1066250 bp. The '*putative *GI' that stretches from 1867500 to 1940000 bp is fragmented into three segments, namely 1872500–1899500 bp, 1903500–1910500 bp and 1911500–1934500 bp (Fig. 3A). In Fig. 3B, the 1D plot for the '*putative *GI' that stretches from 4397500 to 4550000 bp is shown in the lower panel. This '*putative *GI' is fragmented into six segments, namely 4398500–4401500 bp, 4402500–4407500 bp, 4408500–4441500 bp, 4442500–4510500 bp, 4511500–4542500 bp and 4544500–4549500 bp. The third, the fourth and the fifth segments of the above mentioned '*putative *GI' contain mainly phage genes, some pseudo genes and the Vi polysaccharide, which is the major virulence determinant in *S. typhi*. After running the *refinement phase*, the genes excluded from the above mentioned '*putative *GIs' are mainly DNA polymerase III, theta subunit, transcriptional activator protein, putative transcriptional regulator, exodeoxyribonuclease X, ribosome modulation factor (protein E), possible sulfatase regulatory protein, serine/threonine protein phosphatase 1, putative ion and/or amino acid symporter, aminopeptidase N and some hypothetical and conserved hypothetical proteins.

### Performance comparison with other methods

**Figure 4:**
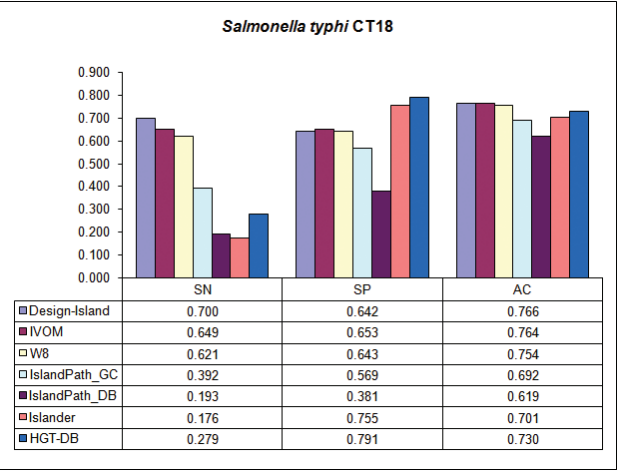
The bar diagram and the corresponding data table for the sensitivity (SN), the specificity (SP) and the accuracy (AC) of Design-Island along with the other methods using a manually curated data set of 1560 putative horizontally transferred genes of *Salmonella typhi *CT18 generated by Vernikos et al. [21].

For performance assessment of *Design-Island*, a dataset of 1560 manually curated putative horizontally transferred genes in *S. typhi *CT18, generated by Vernikos et al. [21] were used. *S. typhi *CT18 is a well-studied prokaryote in terms of its HGT events. Vernikos et al. [21] selected *S. typhimurium *LT2 as a sister lineage to *S. typhi*, and the genome of *E. coli *K12 was chosen as an outgroup of *S. typhi *and *S. typhimurium*. Their main idea was that the genes present in all the three genomes form a set of core genes, while the rest of the genes represent either species or strain specific genes, and thus they may be considered as putative HTGs (keeping in mind the fact that not all the putative HTGs are horizontally acquired; some putative HTGs may arise from gene gain in one genome and gene loss in the other). The sensitivity (SN), the specificity (SP) and the accuracy (AC) of *Design-Island *have been compared with those of six other methods available in the literature, namely W8 [20], IslandPath-GC (based on GC composition), IslandPath-DB (based on di-nucleotide bias) [35], Islander [11], HGT-DB [36] and IVOM [21]. The results are summarized in Fig. 4 accompanied with its data table. The sensitivity of *Design-Island *is the highest (70%) among the methods considered for comparison, the second in the list being IVOM (64.9%). Regarding the accuracy also, the *Design-Island *is in the highest position with an accuracy = 76.6%, and IVOM is in the second position with an accuracy = 76.4%. The third method in the list is W8 with accuracy = 75.4%. The specificity of *Design-Island *(64.2%) is comparable with that of IVOM (65.3%) and W8 (64.3%). However, the specificity of *Design-Island *is low when compared with that of HGT-DB (78.9%) and Islander (75.5%). Note that *Design-Island*, IVOM and W8 predicted a much larger number of putative horizontally transferred genes compared to the number of such horizontally transferred genes predicted by HGT-DB and Islander, and this largely explains the behaviour of different methods in terms of their accuracies as pointed out by earlier authors [21].

Co-ordinates of the detected segments and the percentages of the genome covered by (i) the '*putative *islands' identified in the *first phase *of the algorithm, (ii) genomic segments detected after the *refinement phase *are given in Additional file 1. Further, in the last column of Additional file 1, the genes included in our identified segments along with the percentage of those genes in the entire collection of genes present in the annotated chromosome are presented. The percentages of HTGs identified by different methods are reported in Additional file 2.

Ribosomal proteins and many other highly expressed genes tend to deviate compositionally from the genomic background. However, those genes may have limited mobility, and they may not transfer across species [37]. For this reason, ribosomal proteins, other highly expressed genes with biased compositions and the stretches with heavy loads of ribosomal proteins are excluded from the segments obtained in the *refinement phase *of the algorithm following a similar approach taken by some earlier authors [20,36].

## Conclusion

The method proposed and discussed in this paper is an unsupervised method in the sense that it does not require any training dataset to begin with. The method uses Monte-Carlo statistical tests that are implemented using randomly sampled segments, and normal critical values are used for the test statistic. In many of the earlier methods, no statistical test has been performed, and in some cases, where statistical tests were carried out, the determination of the critical values and the P-values were not adequately justified due to lack of rigorous statistical distribution theory. In *Design-Island*, such difficulties are effectively overcome by using Monte-Carlo statistical tests based on randomly selected segments from a chromosome.

We have carried out an elaborate comparative analysis involving different bacterial genomes, and it demonstrates that the performance of *Design-Island *is often comparable to many other well known methods in terms of their sensitivity, specificity and accuracy. Further, in some cases, *Design-Island *outperforms many of those competing methods.

*Design-Island *can detect new segments of bacterial genomes as parts of some GIs that might have been missed by earlier methods. For example, in the case of *S. typhi *CT18, *Design-Island *has predicted some pathogenic or pathogenicity related genes like putative virulence proteins, putative phage proteins, integrase as horizontally acquired materials that were not detected by earlier methods.

## Availability and requirements

The computer program for *Design-Island *along with a 'readme' file can be downloaded from .

## Abbreviations

GIs: Genomic Islands; DR: Direct repeat; SN: Sensitivity; SP: Specificity; AC: Accuracy; *S. typhi: Salmonella typhi; *SPIs: *Salmonella *Pathogenicity Islands

## Authors' contributions

RC was responsible for development and implementation of the computational analysis as well as drafting of the manuscript. KC advised on data analysis and manuscript preparation. PC advised on development of the methodology, data analysis, designing of the study and manuscript preparation. All authors read and approved the final manuscript.

## Supplementary Material

Additional file 1 Predicted Islands and their gene content in *Salmonella typhi *CT18. The data provided presents genomic segments predicted by *Design-Island *and genes present in those islands. Click here for file

Additional file 2 Percent of genes detected as HTGs in different methods. The data provided presents percentage of genes (among total number of genes) present in the detected islands using different methods. Click here for file
