# Supplementary material for: On detection and assessment of statistical significance of Genomic Islands
Source: BMC Genomics. 2008 Apr 1;9:150. doi: 10.1186/1471-2164-9-150 (PMC2362129; doi:10.1186/1471-2164-9-150)
Supplement: Additional file 2 — Percent of genes detected as HTGs in different methods. The data provided presents percentage of genes (among total number of genes) present in the detected islands using different methods. [file 1471-2164-9-150-S2.pdf]

Percentage of genes detected as HTGs in different methods

Methods	Percentage of genes detected as HTGs
	Salmonella typhi CT18
Design-Island	37.33
IVOM	33.74
W8	32.74
Islandpath-DINUC	23.43
Islandpath-DIMOB	17.24
HGT-DB	11.98
Islander	7.91
